# Bacterial Acquisition in Juveniles of Several Broadcast Spawning Coral Species

**DOI:** 10.1371/journal.pone.0010898

**Published:** 2010-05-28

**Authors:** Koty H. Sharp, Kim B. Ritchie, Peter J. Schupp, Raphael Ritson-Williams, Valerie J. Paul

**Affiliations:** 1 Smithsonian Marine Station at Fort Pierce, Fort Pierce, Florida, United States of America; 2 Mote Marine Laboratory, Sarasota, Florida, United States of America; 3 University of Guam Station, University of Guam, Mangilao, Guam; Northeastern University, United States of America

## Abstract

Coral animals harbor diverse microorganisms in their tissues, including archaea, bacteria, viruses, and zooxanthellae. The extent to which coral-bacterial associations are specific and the mechanisms for their maintenance across generations in the environment are unknown. The high diversity of bacteria in adult coral colonies has made it challenging to identify species-specific patterns. Localization of bacteria in gametes and larvae of corals presents an opportunity for determining when bacterial-coral associations are initiated and whether they are dynamic throughout early development. This study focuses on the early onset of bacterial associations in the mass spawning corals *Montastraea annularis, M. franksi, M. faveolata, Acropora palmata, A. cervicornis, Diploria strigosa*, and *A. humilis*. The presence of bacteria and timing of bacterial colonization was evaluated in gametes, swimming planulae, and newly settled polyps by fluorescence *in situ* hybridization (FISH) using general eubacterial probes and laser-scanning confocal microscopy. The coral species investigated in this study do not appear to transmit bacteria via their gametes, and bacteria are not detectable in or on the corals until after settlement and metamorphosis. This study suggests that mass-spawning corals do not acquire, or are not colonized by, detectable numbers of bacteria until after larval settlement and development of the juvenile polyp. This timing lays the groundwork for developing and testing new hypotheses regarding general regulatory mechanisms that control bacterial colonization and infection of corals, and how interactions among bacteria and juvenile polyps influence the structure of bacterial assemblages in corals.

## Introduction

Coral holobionts are dynamic assemblages consisting of the animal host, symbiotic dinoflagellates (zooxanthellae), bacteria, archaea, fungi, and viruses [1]. Recent research on coral-zooxanthellae associations has focused on how changing environmental conditions, especially increased temperatures, affect dinoflagellate and coral physiology [2], [3], and studies have revealed elegant biochemical mechanisms that regulate species-specific zooxanthellae acquisition by the coral host [4]. In contrast, there is still little known about what controls coral-bacterial interactions and whether true symbioses, or long-term species-specific associations between corals and bacteria, exist. Pandemic outbreaks of diverse bacterial diseases in corals across the globe, among other factors including the demise of herbivorous fish and sea urchins, have facilitated the overgrowth and dominance of macroalgae on reefs and dramatically shifted the ecology of reef habitats [5], [6]. Though identification of pathogens is critical for management and prevention of coral diseases, it is equally important to define and establish a baseline for bacterial diversity associated with healthy corals. A thorough understanding of the dynamic coral-associated bacterial communities, how they establish interactions with coral animals, and the roles they play in coral health is critical for effective reef management.

Recent research has identified diverse, complex bacterial assemblages in and on corals, including the carbonate skeleton, the internal tissue, and the surface mucopolysaccharide layer [7]. Data suggest that bacteria maintain long-term associations with some coral hosts and in some cases may contribute to coral metabolism or provide defense. In some corals, diverse bacterial communities appear to be spatially structured within chemical micro-environments in tissue of branching corals [8], [9]. Cyanobacteria have been shown to fix nitrogen and translocate it to the Caribbean star coral *Montastraea cavernosa* [10], and diverse communities of bacteria with nitrogen fixation genes have been identified in two Hawaiian *Montipora* coral species [11]. Ritchie [12] demonstrated that *Acropora palmata* mucus contains bacteria that can inhibit the growth of known coral pathogens, and some bacteria can induce coral larval settlement [13].

Bacteria have also been implicated in declining coral health. Changes in bacterial communities within coral mucus correlated with shifts in coral disease [14], [15], [16], and altered gene expression in mucus-associated bacteria was shown to be involved in coral bleaching [17], [18]. Recent investigations of white band disease in Caribbean corals suggest that components of agricultural and industrial runoff impact coral health by altering growth rates and metabolism of coral-associated microbes [19]. Overall, the role of bacterial communities in coral health appears to be variable; the extent to which species-specific bacterial associations with corals persist across geographical and temporal gradients is unknown; and the physiological and genetic basis for the maintenance of specific associations among host populations from one generation to the next remains undetermined.

The Caribbean corals in this study, *Montastraea faveolata*, *Montastraea annularis*, *Montastraea franksi*, *Acropora palmata*, *Acropora cervicornis*, and *Diploria strigosa*, release gametes in seasonal mass spawning events, and species-specific external fertilization occurs in the water column. Recent research shows that three cryptic *Montastraea* species, *Montastraea annularis*, *M. faveolata*, and *M. franksi*, live sympatrically in both Panama and the Bahamas, but temporal and spatial mechanisms can prevent cross-specific hybrid fertilization among these species [20], [21]. All six species in this study are hermaphroditic, and eggs and sperm from multiple individuals are simultaneously broadcast on the same lunar phases for external fertilization [22], [23]. Once fertilization occurs in the water column, embryos develop into ciliated non-feeding planula larvae with two cell layers. The planulae swim in the water column for a variable time period, ranging from approximately 96–144 hours [23], [24]. Upon selection and attachment to suitable substrata, the larvae undergo metamorphosis into a juvenile polyp [25]. Bacterial communities associated with corals during early developmental stages remain largely uncharacterized in most coral species, with the exception of the species *Pocillopora meandrina*, a spawning coral that transmits zooxanthellae via its eggs and appears to acquire bacteria during planula larval stages [26].

The larvae and subsequent early developmental stages of stony corals present a unique opportunity for microbiology research. In contrast to their adult counterparts, the early life stages have not accumulated a high bacterial load from the surrounding environment or by feeding. Research on these early life-history stages offers the ability to “weed out” microbes that are incidentally in or on the adult tissue and mucus layer and to determine the timing of onset of bacterial associations. Identification of inherited bacteria could reveal bacteria that are potentially significant to the survival and fitness of the larvae or host. Vertical symbiont transmission (trans-ovarian inheritance) has been documented in numerous marine invertebrate-bacterial associations, including but not limited to bryozoans [27], sponges [28], [29], [30], ascidians [31], and bivalves [32], [33], [34], [35]. Species-specific transmission suggests a history of selection for the maintenance of certain bacteria over evolutionary time, and vertical transmission of specific symbionts is often reflected by highly co-evolved host-symbiont phylogenies [36]. However, horizontal bacterial acquisition is also significant to highly specific symbioses and can be regulated by elegant, specific biochemical mechanisms, as is the case in the well-described association between the Hawaiian bobtail squid *Euprymna scolopes* and the bioluminescent *Vibrio fischeri* [37]. Characterizing the onset of bacterial-coral associations may reveal mechanisms by which bacterial colonization and proliferation within coral tissue are regulated. The aim of this study is to determine whether mass spawning corals initiate associations with bacteria via inheritance from parent colonies or through horizontal acquisition of bacteria from the surrounding seawater.

## Materials and Methods

Belize Fisheries Department provided permits and facilitated the research on coral larvae at Carrie Bow Cay, Belize. Coral gametes in the Florida Keys were collected under permit FKNMS-2006-025.

### Gamete collection

**Figure 1:**
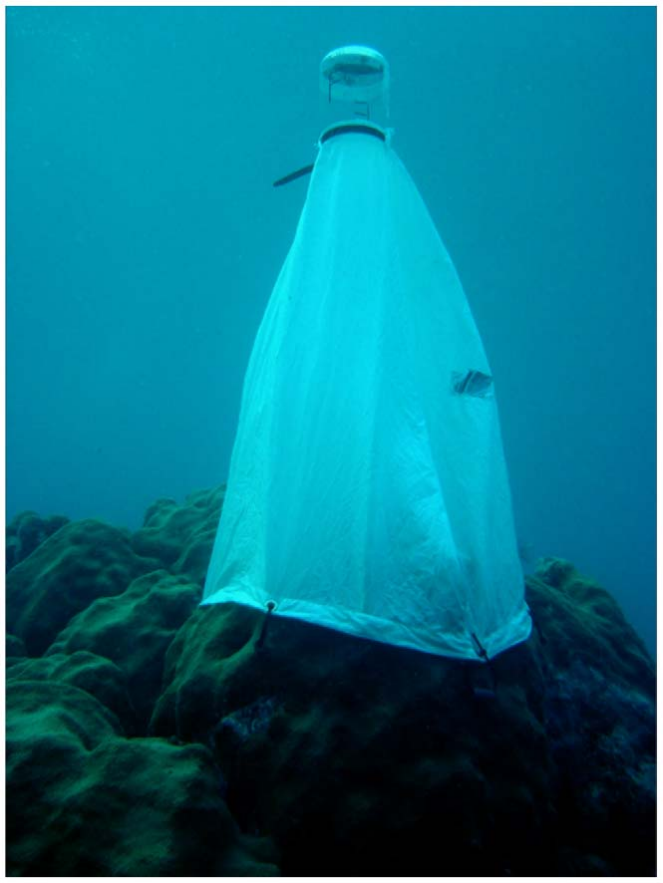
Spawning tent used to collect gametes from coral colonies. Photo: Erich Bartels.

A summary of the dates and locations of spawning events from which gametes were collected for this study is presented in Table 1. Gametes were collected from Looe Key Buoy #21 during an *A. palmata* spawning event in August 2007 and a *M. faveolata* spawning event in September 2007. *Diploria strigosa* gametes were collected from colonies at Grecian Rocks reef in Key Largo during a spawning event in September 2007. Gametes were obtained from spawning colonies with non-invasive nylon mesh collection tents attached to polypropylene collection jars, such as the apparatus shown in Figure 1.

**Table 1:** A list of the locations and timing of all gamete collections described in this study.

Species	Spawning Year	Collection Location
*Acropora cervicornis*	August 2005	Carrie Bow Cay, Belize
		16°48.18′N, 88°04.93′W
*Montastraea franksi*	September 2006	Carrie Bow Cay, Belize
		16°48.18′N, 88°04.93′W
*Montastraea annularis*	September 2006	Carrie Bow Cay, Belize
		16°48.18′N, 88°04.93′W
*Montastraea faveolata*	September 2006	Carrie Bow Cay, Belize
		16°48.18′N, 88°04.93′W
*Acropora palmata*	September 2006	Carrie Bow Cay, Belize
		16°48.18′N, 88°04.93′W
*Acropora palmata*	August 2007	Looe Key, Florida Keys, US
		24°32.75′ N, 81°24.35′ W
*Montastraea faveolata*	September 2007	Looe Key, Florida Keys, US
		24°32.75′ N, 81°24.35′ W
*Diploria strigosa*	September 2007	Grecian Rocks, Key Largo, FL
		25°06.91′N, 080°18.20′W
*Acropora humilis*	August 2007	Pago Bay, Guam
		13°42.67′N, 144°79.86′E

In the Belize 2005 and 2006 collections, gametes were collected from *A. palmata* colonies with non-invasive nylon mesh collection tents and polypropylene collection jars, similar to the design used for gamete collection in the Florida Keys. Small colonies of *A. cervicornis*, *M. franksi, M. faveolata*, and *M. annularis* were collected from the reefs surrounding Carrie Bow Cay, Belize and were placed on submerged racks where they were maintained for several days. Each night, the colonies were placed in 5-gallon buckets full of freshly collected reef water for several hours, and upon gamete release, gametes were collected from the buckets via glass Pasteur pipets. Every night after gamete collection was complete, the colonies were returned to the submerged racks. After gamete collection, coral colonies were reattached on their reef using Splash Zone underwater epoxy (Z-Spar).

Fertilization was achieved by mixing gametes from separate colonies in buckets of reef water, and the resulting embryos developed into swimming larvae in flow-through reef water (Belize), or fresh, filtered reef water (0.45 µm filtered Looe Key reef water) that was changed twice per day (Florida Keys). Samples were fixed for microscopy (see below) at sequential time points including gametes (t = 0 h) and larvae at subsequent 24 h intervals until settlement. In the 2007 *M. faveolata* collection from Florida, some larvae were reared in the above conditions until 96 h post-gamete release. At 96 h, swimming larvae were moved to 500 ml beakers containing 0.45 µm filtered reef water and untreated glass microscopy slides. A small percentage of the larvae settled and metamorphosed on the microscopy slides, and those settled individuals were fixed for microscopy (as described below) after attachment. Larvae were classified as metamorphosed if they had transformed into a juvenile polyp. A few days after metamorphosis, carbonate skeleton deposition was observed.

In the 2007 Guam collection, *Acropora humilis* gametes were collected from laboratory colonies. Colonies (15–20 cm diameter in size) were collected one week before the spawning event from the fore reef slope (2–5 m depth) in Pago Bay, Guam. Colonies were maintained in 72 L tanks (1 per tank) with unfiltered flow-through seawater. For gamete collection colonies were kept without running seawater and aeration only. Egg and sperm bundles from different colonies were mixed upon release, and developing larvae were maintained in 13 L of filtered seawater (200 µm), which was changed twice daily. After 8 days, larvae were pipetted into wax-coated 15 ml petri dishes, each of which contained a glass microscopy slide. One to three small pieces of the crustose coralline alga (CCA) *Hydrolithon* sp. were placed on the glass slides to facilitate larval settlement because only occasional settlement was observed in preliminary experiments without CCA added to the petri dishes. Typically, 3–5 larvae settled on the glass slide, which produced a sufficient number of settled polyps on the glass slides for FISH experiments at distinct time intervals.

### Fixation of Larvae for FISH

Gametes and swimming larvae were rinsed three times in sterile filtered seawater (0.22 µm), fixed in paraformaldehyde (4% in buffer: 20 mM K_2_HPO_4_, 0.5 M NaCl, pH 7.4) overnight at 4°C, and transferred to 70% ethanol for long-term storage at −20°C. To fix polyps that settled on glass slides, the slides were dipped three times in separate 50 ml polypropylene tubes of sterile filtered seawater (0.22 µm). The slides were then submerged in 4% paraformaldehyde in 50 ml polypropylene tubes and fixed overnight at 4°C. The 4% paraformaldehyde was discarded, and the tube was filled with 70% ethanol for long-term storage at −20°C.

### FISH and Microscopy Analysis

FISH was performed on fixed whole gametes or larvae in microfuge tubes, or settled polyps on microscopy slides with hybridization buffer (0.9 M NaCl, 20 mM Tris-HCl [pH 7.4], 0.01% sodium dodecyl sulfate) containing 35% percent formamide. Samples were probed with a suite of general eubacterial probes, added in equimolar amounts: EUB338I (5′-GCTGCCTCCCGTAGGAGT-3′); EUB338II (5′-GCA GCC ACC CGT AGG TGT-3′), and EUB338III (5′-GCT GCC ACC CGT AGG TGT-3′) [38]. Negative control samples were probed with the negative control probe, NONEUB (5′-ACT CCT ACG GGA GGC AGC-3′) [38]. Probes were ordered as CY3-end labeled oligonucleotides (Integrated DNA Technologies, Coralville, IA). For each set of FISH reactions, a sample in which the bacterial content and location was known was probed with the EUB338 probe suite as a positive control for probe and reagent quality. All probes were added to hybridization buffer at a final concentration of 5 ng/µl. After 2 h hybridization at 46°C, the hybridization buffer was removed from the samples. Samples were incubated in wash buffer (0.7 M NaCl, 20 mM Tris-HCl [pH 7.4], 50 mM EDTA, 0.01% sodium dodecyl sulfate) for 20 minutes at 48°C. The samples were rinsed with Milli-Q water and mounted in VectaShield (*Vector Labs*, Burlingame, CA). Slides were visualized on an LSM510 laser scanning confocal microscope (Zeiss, Jena, Germany). Approximately 50–100 individuals of each stage were imaged, depending on sample availability.

## Results

**Figure 2:**
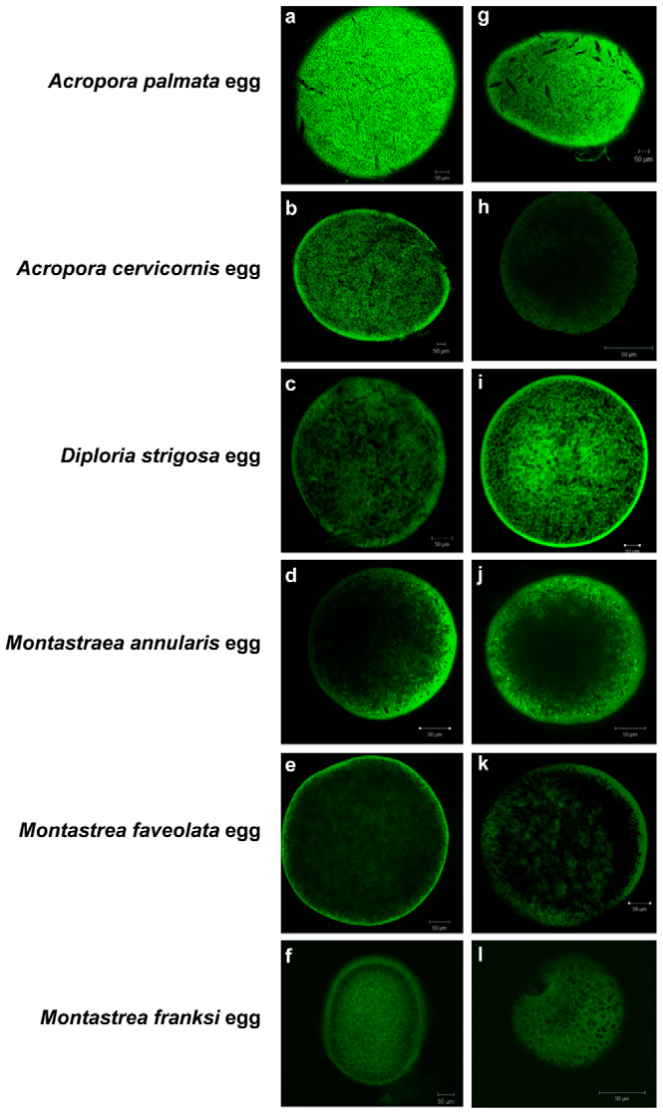
General eubacterial FISH in gametes of six Caribbean coral species. FISH visualization of the CY3-labeled general eubacterial probe suite (EUB338I/EUB338II/EUB338III) on eggs from the six species of Caribbean scleractinian corals, *Montastraea annularis, M. franksi, M. faveolata, Acropora palmata, A. cervicornis,* and *Diploria strigosa*. Panels a–f, EUB338; g–l, NONEUB (negative control). No bacterial signal is visible in or on the eggs of any of the species.

Eggs and sperm were both examined for the presence of bacterial cells, indicated by fluorescent signal from the eubacterial FISH probe suite (EUB338) (Figure 2a–f). No bacteria were detected in the sperm (not shown) or eggs of six different coral species. Though the background autofluorescence in the eggs is high, there is little difference between signal from the probed samples and the controls for non-specific probe binding (NONEUB probe) (Figure 2g–l).

**Figure 3:**
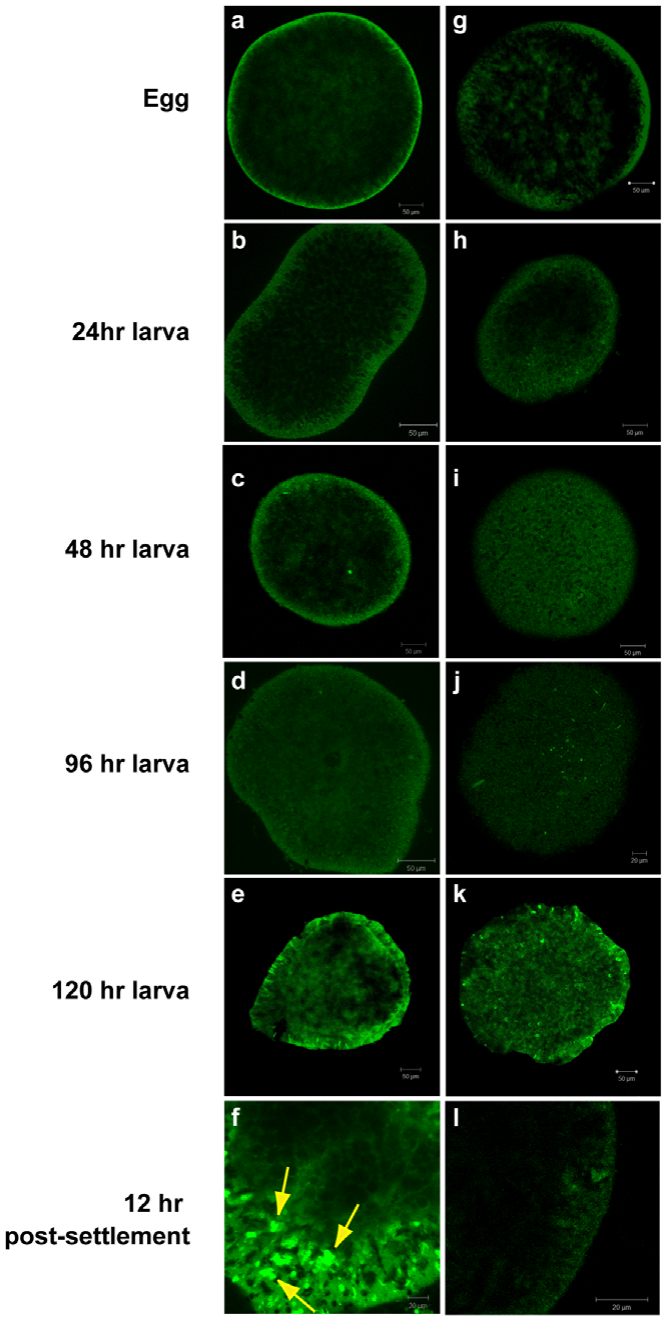
General eubacterial FISH in early development of *Montastraea faveolata*. FISH visualization of the CY3-labeled general eubacterial probe suite (EUB338I/EUB338II/EUB338III) on *Montastraea faveolata* eggs, larvae, and settled juvenile polyps. Panels a–f, EUB338; panels g–l, NONEUB (negative control). No signal is visible in stages up to 120 h (panels a–e), but 24 h after settlement, the surface of the polyps is colonized by bacteria (panel f; yellow arrows). The 120 h planulae show evidence of developing nematocysts, which is presented as non-specific probe binding in both the EUB338 treatment (panel e) and the NONEUB negative probe control treatment (panel k).

EUB338 FISH results from *M. faveolata* early development, a time series of stages spanning from newly released eggs to 24 h post-settlement (Figure 3a–f), show that bacteria were not detectable in the eggs or in *M. faveolata* planulae through 120 h post-release. The nematocysts are apparent on the surface of the polyps in both the EUB338 probe- and NONEUB probe-treated samples (Figure 3e,k), indicating non-specific probe binding to nematocysts. Bacteria were observed on the surface of the *M. faveolata* recruits, which had attached and then developed for 24 h into juvenile polyps (Figure 3f). Negative controls probed with the NONEUB probe are shown in Figure 3g–l, corresponding to the stages probed with the EUB338 suite.

**Figure 4:**
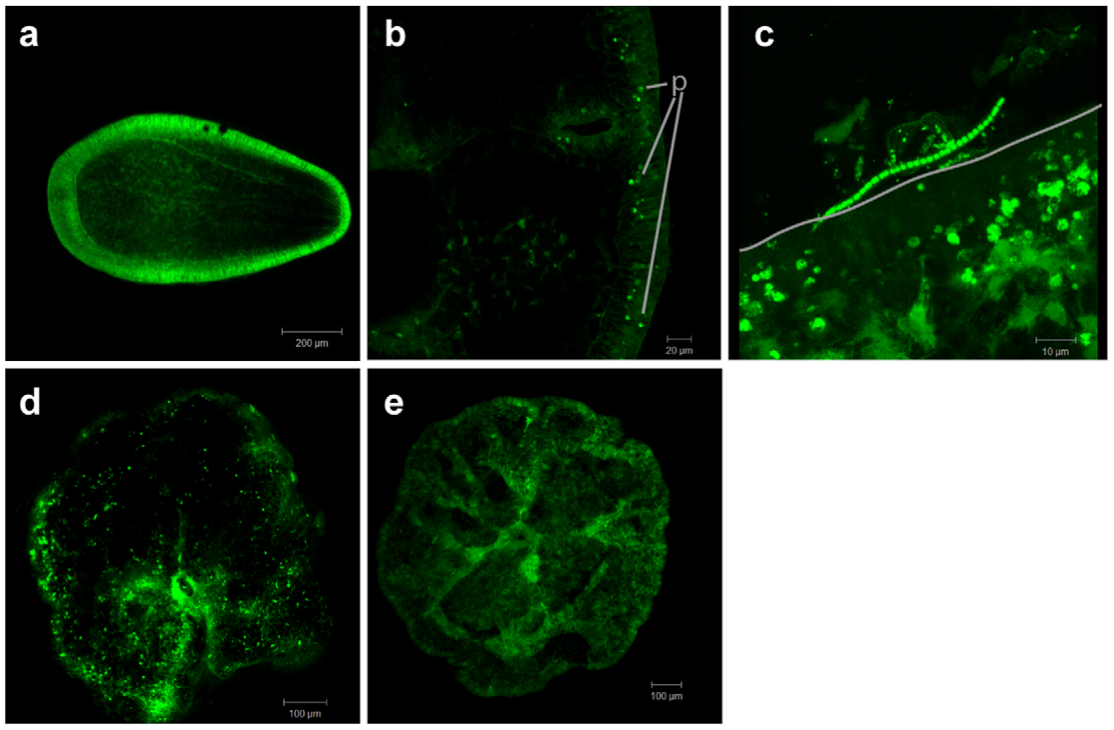
Bacteria in juvenile *Acropora humilis* polyps. FISH visualization of signal from the CY3-labeled general eubacterial probe suite (EUB338I/EUB338II/EUB338III) on early stages of *Acropora humilis*. Panel a, a cross-section of a 129 h planula larva probed with the CY3-EUB338 suite, showing no probe signal. Panel b, a cross-section of a settled polyp, showing autofluorescent pigment granules (p) in the epidermis of a juvenile polyp but no bacterial cells hybridizing to the suite of general eubacterial probes. Panel c, a three-dimensional projection showing the same *A. humilis* polyp from the settlement substrate to the top of the polyp surface. The line delineates the edge of the polyp on the settlement slide. Several different morphotypes of CY3-EUB338-hybridized bacterial cells (b) are visible on the glass settlement slide, but the interior and the surface of the polyp do not contain any bacterial signal. Panel d, the surface of a juvenile *A. humilis* polyp with bacterial cells hybridizing with the CY3-EUB338 probe suite across the polyp surface. Panel e, the negative control (CY3-NONEUB) shows no signal on an *A. humilis* juvenile.

No bacteria were detected in *A. humilis* stages up through 129 h after release, immediately prior to settlement (Figure 4a). Figure 4b shows a cross-section of a 12 h post-settlement juvenile polyp probed with EUB338. In this stage, there are no bacterial cells visible within the epidermis or gastrodermis. The interface between the same 12 h polyp and the settlement substratum (glass microscopy slide), shown in a three-dimensional projection (Figure 4c), shows abundant bacterial cells and diverse cell morphologies outside of the polyp, but bacteria are neither present on the coral polyp surface nor in the polyp interior. Figure 4d shows an *A. humilis* juvenile polyp, 24 hours post-settlement, and EUB probe signal is visible across the polyp surface. In the negative probe control (Figure 4e), no signal is seen on the polyp surface.

## Discussion

The corals in this study represent several different spawning species and include corals collected from locations spanning thousands of miles and four years of collection. None of these corals transmitted bacteria to their offspring via gametes. In the spawning corals examined in this study, throughout the 5-day swimming larval period, bacteria did not appear to be taken up by planulae, nor were they detectable on the planula surface. Only after the larvae settled and metamorphosed were there detectable numbers of bacteria in the corals, even though the coral larvae were exposed to seawater containing bacteria during their development. This suggests that either bacteria were unable to colonize the larvae in high numbers during swimming stages, or that some change occurred in the coral after settlement that either allowed or decreased the inhibition of bacterial colonization.

One possible explanation for the observed timing of bacterial colonization in the juvenile coral polyps is that eggs and newly developed larvae contain chemical defenses that are lethal to bacteria. While the presence of antimicrobial compounds for protection of egg masses in mollusks is well documented [39], [40], Marquis and colleagues [41] showed that out of eleven Pacific coral species screened, eggs of only one species, *Montipora digitata*, contained antimicrobial compounds. Because a crude extract of *M. digitata* eggs inhibited the growth of only three out of ninety-three tested bacterial strains, it seems unlikely that antibacterial compounds in the eggs and larvae of the corals in our study explain the delayed bacterial colonization.

To date, a mechanistic link between developmental changes in corals and recruitment of bacteria has not been identified. Whether compound production by the host plays a role in attracting bacteria is unclear. Morphogenesis in the bobtail squid *Euprymna scolopes* results in tissue-localized shifts in gene expression and production of specific receptors that induce symbiotic attachment and infection [37]. It is possible that corals may exhibit stage-specific gene expression, attracting bacteria during post-settlement stages. It has been demonstrated that the adhesion of the pathogenic bacterium *Vibrio shiloi* to a β–D-galactoside-containing receptor in the mucus of the coral *Oculina patagonica* is dependent on the presence of actively photosynthesizing zooxanthellae [42], suggesting that the zooxanthellae play a role in bacterial adhesion. Another study suggested that coral and zooxanthellae metabolic contributions to coral mucus may select for specific functional groups of bacteria [43]. Further research exploring the timing of *Symbiodinium* acquisition relative to the timing of bacterial acquisition may provide insight into whether zooxanthellae are involved in bacterial colonization of juvenile corals. In addition, further histological investigation of bacterial presence in coral recruits is required to determine when and how bacteria enter coral tissues.

These results describe the initial onset of bacterial-coral associations in a wide range of coral hosts. It has not been determined whether acquisition of bacteria by corals from the water column is a selective process or whether bacterial colonization of juvenile corals is simply opportunistic in nature. Apprill *et al.* (2009) suggested that the spawning coral *Pocillopora meandrina* acquired a specific bacterial associate, belonging to the alpha-proteobacterial subdivision, but to date, that is the only documented specific association between bacteria and early life stages of a coral. It is currently unknown whether corals select certain bacteria from the surrounding seawater during these vulnerable early life stages, or how juvenile corals will respond to alterations in seawater quality and subsequent changes in seawater bacterial communities. Understanding the factors that control the timing and specificity of bacterial colonization will provide insight into communication among the multiple partners in the coral holobiont and identify significant determinants in host vulnerability to bacterial disease and changing marine environments.
